# Supplementary material for: Vegetative cells may perform nitrogen fixation function under nitrogen deprivation in Anabaena sp. strain PCC 7120 based on genome-wide differential expression analysis
Source: PLoS One. 2021 Mar 4;16(3):e0248155. doi: 10.1371/journal.pone.0248155 (PMC7932525; doi:10.1371/journal.pone.0248155)
Supplement: S1 File — (DOC) [file pone.0248155.s001.doc]

**S1.1 library preparation**

The extracted RNA was first checked using 1% agarose gels before the quality of the RNA was assessed using an Agilent 2100 Bioanalyzer system (Agilent 2100 Bioanalyzer . RNA 6000

Nano kit 5067-1511, CA, USA). RNA with a RIN value greater than 8.0 was used to produce a transcriptome library. Illumina Ribo-Zero Magnetic Kit was used to remove the rRNA from the total RNA sample according to the manufacturer’s instructions. RNA sequencing libraries were generated using an NEBNext Ultra Directional RNA library prep kit for Illumina (NEB, USA) following the manufacturer’s instructions. Library quality was assessed on an Agilent Bioanalyzer 2100 system(Agilent 2100 Bioanalyzer, Agilent, 2100; Agilent High Sensitivity DNA Kit, Agilent, 5067-4626).The methods of quantitative of library were Pico green and fluorescence spectrophotometer (Quantifluor-ST fluorometer, Promega, E6090; Quant-iT PicoGreen dsDNA Assay Kit, Invitrogen, P7589), and the quality control of the enrichment of the PCR fragments and validation of the size and distribution of DNA fragments in the library were conducted using Agilent 2100 (Agilent 2100 Bioanalyzer, Agilent, 2100; Agilent High Sensitivity DNA Kit, Agilent, 5067-4626). The samples mixed after homogenization to 10 nM, then diluted gradually and quantitated to 4~5pM for Illumina sequencing to construct the multiplexed DNA libraries.

**S1.2 Mapping reads to the reference genome and normalized gene expression**

The quality information of raw data in FASTQ format was calculated, and then the raw data were filtered using Cutadapt (v1.15) software. Then, high-quality clean data were obtained by removing reads containing adapters, reads containing poly-N, and low-quality reads. The remaining clean reads were mapped to the annotated genome of *Nostoc* sp. PCC 7120 genome (https://www.ncbi.nlm.nih.gov/genome/genomes/13531. BioSample = SAMD00061094, BioProject = PRJNA244; BioSample = SAMN10102199, BioProject = PRJNA492407) by using Bowtie 2 software based on the local alignment algorithm.
