# Supplementary material for: Vegetative cells may perform nitrogen fixation function under nitrogen deprivation in Anabaena sp. strain PCC 7120 based on genome-wide differential expression analysis
Source: PLoS One. 2021 Mar 4;16(3):e0248155. doi: 10.1371/journal.pone.0248155 (PMC7932525; doi:10.1371/journal.pone.0248155)
Supplement: S1 Fig — (DOC) [file pone.0248155.s002.doc]

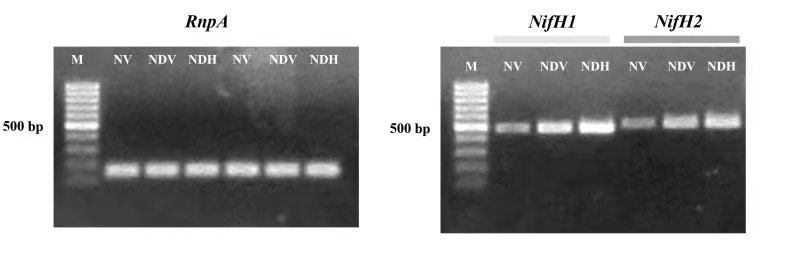


S1 Fig. *NifH1* and *nifH2* gene expression analysis by RT-PCR
*RnpA* was used as internal reference gene, and RT-PCR primers were designed according to the coding sequences of *NifH1* and *NifH2*. The product sizes of *NifH1* and *NifH2* amplified by RT-PCR were 477 bp and 513 bp respectively. The PCR reaction conditions were as follows: denaturation at 94 ℃ for 5 min; denaturation at 94 ℃ for 30 s, renaturation at 58 ℃ for 45 s, extension at 72 ℃ for 40 s, 26 cycles; 72 ℃ for 10 minutes; stored at 4 ℃. PCR amplified products were electrophoretic on 1% agarose gel. Primers sequences were listed as follows.

*RnpA*-F: 5' -TTGCGAGCATTAAAACCGTC- 3', *RnpA*-R: 5’- TACGCACCACCGCCCTCT -3';

*NifH1*-F: 5' -ATTGGGTGACGTTGTATGTGGT-3', *NifH1*-R: 5'-CCGATGATTTCAGAGTGCTTG-3';

*NifH2*-F: 5' -TATCACCGCCATCAACTTCTTAG-3', *NifH2*-R: 5'-GAATACCGAACTCAATCAGCAACT-3'.
