# Supplementary material for: Vegetative cells may perform nitrogen fixation function under nitrogen deprivation in Anabaena sp. strain PCC 7120 based on genome-wide differential expression analysis
Source: PLoS One. 2021 Mar 4;16(3):e0248155. doi: 10.1371/journal.pone.0248155 (PMC7932525; doi:10.1371/journal.pone.0248155)
Supplement: S1 Table — (DOCX) [file pone.0248155.s003.docx]

S1 Table. RNA-seq reads of *Anabaena* sp. strain PCC 7120 mapped to reference genome

| **Sample** | **Clean data (Bp)** | **Useful Reads** | **Total Mapped Reads** | **Uniquely Mapped Reads** | | **Multiple Mapped Reads** | |
| --- | --- | --- | --- | --- | --- | --- | --- |
|  |  |  |  | **Number** | Percentage  (%) | **Number** | Percentage  (%) |
| NV1 | 4368142798 | 28,928,098 | 27,185,644 | 25,926,965 | 95.37 | 1,258,679 | 4.63 |
| NV2 | 4114895866 | 27,250,966 | 25,393,272 | 24,021,334 | 94.60 | 1,371,938 | 5.40 |
| NV3 | 4019568962 | 26,619,662 | 24,778,007 | 23,440,231 | 94.60 | 1,337,776 | 5.40 |
| NDV1 | 3852369380 | 25,512,380 | 24,689,173 | 23,356,858 | 94.60 | 1,332,315 | 5.40 |
| NDV2 | 3893397288 | 25,784,088 | 24,919,251 | 23,546,778 | 94.49 | 1,372,473 | 5.51 |
| NDV3 | 4102228476 | 27,167,076 | 26,242,699 | 24,698,152 | 94.11 | 1,544,547 | 5.89 |
| NDH1 | 4074845834 | 26,985,734 | 25,357,896 | 24,024,527 | 94.74 | 1,333,369 | 5.26 |
| NDH2 | 4013121866 | 26,576,966 | 24,962,871 | 23,773,446 | 95.24 | 1,189,425 | 4.76 |
| NDH3 | 4275516076 | 28,314,676 | 26,533,459 | 25,198,586 | 94.97 | 1,334,873 | 5.03 |

Note: Useful reads: the total number of sequences used for comparison; Total mapped reads: total read number that mapped to reference genome sequences; Unique mapped reads: number and percentage of read that is aligned to one location in reference genome; Multiple Mapped Reads: number and percentage of read that is aligned to multiple locations in reference genome.
