# Supplementary material for: Vegetative cells may perform nitrogen fixation function under nitrogen deprivation in Anabaena sp. strain PCC 7120 based on genome-wide differential expression analysis
Source: PLoS One. 2021 Mar 4;16(3):e0248155. doi: 10.1371/journal.pone.0248155 (PMC7932525; doi:10.1371/journal.pone.0248155)
Supplement: S2 Table — (DOCX) [file pone.0248155.s004.docx]

S2 Table. Distribution statistics results of RNA-Seq mapped events for all reads

| Sample | InterGene | | Gene | | mRNA | | rRNA | |
| --- | --- | --- | --- | --- | --- | --- | --- | --- |
|  | Number | Percentage  (%) | Number | Percentage  (%) | Number | Percentage  (%) | Number | Percentage  (%) |
| NV1 | 1564799 | 5.76 | 25,620,845 | 94.24 | 20,639,528 | 80.56 | 199,880 | 0.78 |
| NV2 | 1447817 | 5.70 | 23,945,455 | 94.30 | 19,670,956 | 82.15 | 304,150 | 1.27 |
| NV3 | 1356817 | 5.48 | 23,421,190 | 94.52 | 19,156,565 | 81.79 | 297,991 | 1.27 |
| NDV1 | 1700302 | 6.89 | 22,988,871 | 93.11 | 17,809,363 | 77.47 | 354,168 | 1.54 |
| NDV2 | 1762310 | 7.07 | 23,156,941 | 92.93 | 17,954,725 | 77.53 | 379,944 | 1.64 |
| NDV3 | 1881431 | 7.17 | 24,361,268 | 92.83 | 19,218,496 | 78.89 | 485,137 | 1.99 |
| NDH1 | 1821772 | 7.18 | 23,536,124 | 92.82 | 18,503,273 | 78.62 | 269,910 | 1.15 |
| NDH2 | 1752157 | 7.02 | 23,210,714 | 92.98 | 17,169,082 | 73.97 | 192,388 | 0.83 |
| NDH3 | 1858233 | 7.00 | 24,675,226 | 93.00 | 18,141,668 | 73.52 | 260,265 | 1.05 |

Note: Intergene, gene, mRNA and rRNA: the total number and proportion of reads compared to the intergenic region, gene, mRNA and rRNA respectively.
