# Supplementary material for: Vegetative cells may perform nitrogen fixation function under nitrogen deprivation in Anabaena sp. strain PCC 7120 based on genome-wide differential expression analysis
Source: PLoS One. 2021 Mar 4;16(3):e0248155. doi: 10.1371/journal.pone.0248155 (PMC7932525; doi:10.1371/journal.pone.0248155)
Supplement: S3 Table — (DOCX) [file pone.0248155.s005.docx]

S3 Table. The DEGs encoding PS I, PS II in three comparison pairs

| Gene ID | ORF ID | log_2_FoldChange  (NV vs. NDV) | log_2_FoldChange  (NV vs. NDH) | log_2_FoldChange  (NDV vs. NDH) | Length  bp | Gene annotation |
| --- | --- | --- | --- | --- | --- | --- |
| gene5103 | alr5154 | -0.67 | 0.04 | 0.70 | 2258 | *psaA* (PS-I) |
| gene5264 | alr5314 | **-2.36 *** | **-1.49*** | 0.87 | 2228 | *psaB* (PS-I) |
| gene5104 | alr5155 | -0.73 | 0.05 | 0.77 | 2225 | *psaB* (PS-I) |
| gene4262 | alr4290 | -0.09 | 0.28 | 0.37 | 1055 | *psaD* (PS-I) |
| gene3438 | asr3463 | -0.48 | -0.19 | 0.28 | 245 | *psaC* (PS-I) |
| gene319 | all0329 | -0.25 | 0.38 | 0.62 | 419 | *psaD* (PS-I) |
| gene4289 | asr4319 | -0.26 | 0.12 | 0.37 | 212 | *psaE* (PS-II) |
| gene102 | all0109 | 0.06 | 0.45 | 0.39 | 494 | *psaF* (PS-I) |
| gene101 | asl0108 | 0.10 | 0.27 | 0.16 | 149 | *psa*J (PS-I) |
| gene4729 | asr4775 | **-1.00 *** | -0.27 | 0.72 | 260 | *psaK* (PS-I) |
| gene100 | all0107 | 0.02 | 0.41 | 0.38 | 518 | *psaL* (PS-I) |
| gene4618 | asr4657 | -0.79 | -0.44 | 0.35 | 122 | *psaM* (PS-I) |
| gene1259 | asr1283 | 0.07 | 0.61 | 0.53 | 134 | *psaX* (PS-I) |
| gene3973 | all4001 | -0.68 | **1.75*** | **2.42 *** | 1034 | *isiA* (PS-II) |
| gene3709 | alr3742 | -0.14 | -0.02 | 0.11 | 1082 | *psbA* (PS-II) |
| gene4819 | alr4866 | -0.15 | 0.36 | 0.49 | 1082 | *psbAI* (PS-II) |
| gene3695 | alr3727 | -0.41 | 0.67 | **1.08 *** | 1082 | *psbAII* (PS-II) |
| gene4554 | alr4592 | -0.20 | 0.95 | **1.14 *** | 1082 | *psbAIII* (PS-II) |
| gene3547 | all3572 | -0.15 | **1.16*** | **1.31 *** | 1082 | *psbAIV* (PS-II) |
| gene132 | all0138 | -0.34 | 0.21 | 0.54 | 1529 | *psbB* (PS-II) |
| gene4510 | alr4548 | -0.08 | 0.8 | 0.87 | 1055 | *psbD* (PS-I) |
| gene4263 | alr4290 | -0.71 | -0.15 | 0.55 | 1379 | *psbD* (PS-II) |
| gene824 | asl0846 | -0.42 | -0.31 | 0.1 | 188 | *psbH* (PS-II) |
| gene3814 | asr3848 | -0.55 | 0.12 | 0.37 | 122 | *psbJ* (PS-II) |
| gene864 | asl0885 | 0.50 | 0.06 | -0.44 | 137 | *psbK* (PS-II) |
| gene3813 | asr3847 | 0.34 | 0.41 | 0.38 | 122 | *psbL* (PS-II) |
| gene862 | asl0883 | 0.62 | 0.37 | -0.24 | 116 | *psbM* (PS-II) |
| gene825 | asr0847 | **-1.30 *** | -0.09 | **1.19 *** | 131 | *psbN* (PS-II) |
| gene131 | asl0137 | 0.65 | 0.72 | 0.07 | 107 | *psbT* (PS-II) |
| gene1194 | alr1216 | 0.01 | 0.13 | 0.11 | 428 | *psbU* (PS-II) |
| gene780 | all0801 | -0.68 | -0.26 | 0.4 | 335 | *psbW* (PS-II) |
| gene922 | asr0941 | 0.01 | -0.05 | -0.06 | 119 | *psbX* (PS-II) |

Note: Log_2_FC, Log_2_Fold change. *, differently expressed gene with 1 < |Log_2_FC| < 3; **, highly differently expressed gene with Log_2_FC ≥ 3. In three paired comparisons, X vs. Y refers to Y/X.
