# Supplementary material for: Vegetative cells may perform nitrogen fixation function under nitrogen deprivation in Anabaena sp. strain PCC 7120 based on genome-wide differential expression analysis
Source: PLoS One. 2021 Mar 4;16(3):e0248155. doi: 10.1371/journal.pone.0248155 (PMC7932525; doi:10.1371/journal.pone.0248155)
Supplement: S4 Table — (DOCX) [file pone.0248155.s006.docx]

S4 Table. The DEGs encoding nitrogenase genes in three comparison pairs

| Gene ID | ORF ID | log_2_FoldChange  (NV vs. NDV) | log_2_FoldChange  (NV vs. NDH) | log_2_FoldChange  (NDV vs. NDH) | Gene Length | Gene Name |
| --- | --- | --- | --- | --- | --- | --- |
| gene1427 | all1454 | **5.55**** | **8.83**** | **3.28**** | 1442 | *NifD* |
| gene1411 | all1437 | **3.93**** | **6.64**** | **2.70*** | 1334 | *NifN* |
| gene853 | alr0874 | -0.95 | **1.11*** | **2.06*** | 893 | *NifH2* |
| gene1410 | all1436 | **5.10**** | **7.80**** | **2.69*** | 413 | *NifX* |
| gene2517 | all2531 | 0.39 | -0.89 | **-1.29*** | 506 | *NifX* |
| gene1428 | all1455 | **5.80**** | **9.01**** | **3.20**** | 887 | *NifH1* |
| gene1415 | all1454 | **7.19**** | **10.79**** | **3.60**** | 86 | *NifD* |
| gene1414 | all1440 | **5.75**** | **9.02**** | **3.26**** | 1538 | *NifK* |
| gene1430 | all1457 | **2.50*** | **5.53**** | **3.02**** | 1202 | *NifS* |
| gene1490 | all1517 | **1.97*** | **4.68**** | **2.70*** | 1427 | *NifB* |
| gene1429 | all1456 | **1.68*** | **4.16**** | **2.48*** | 902 | *NifU* |
| gene2946 | alr2968 | 0.55 | **1.24*** | 0.69 | 1130 | *NifV2* |
| gene1887 | alr1911 | **1.38*** | **1.21*** | -0.18 | 3536 | *NifJ* |
| gene1412 | all1438 | **3.20**** | **6.11**** | **2.90*** | 1442 | *NifE* |
| gene1407 | all1433 | Inf | Inf | **2.87*** | 317 | *NifW* |
| gene1381 | alr1407 | **2.05*** | **2.89*** | 0.84 | 1133 | *NifV1* |
| gene1382 | asr1408 | Inf | Inf | **-1.24*** | 287 | *NifZ* |
| gene1284 | asr1309 | 0.77 | **1.26*** | 0.49 | 230 | *NifU2* |
| gene1416 | alr1442 | 0.51 | 0.69 | 0.18 | 1301 | *NifD* |
| gene1489 | all1516 | **3.55**** | **6.18**** | **2.63*** | 323 | *FKXN* |
| gene673 | all0688 | **4.54**** | **6.18**** | **1.63*** | 962 | *NifE* |
| gene672 | all0687 | **3.27**** | **4.72**** | **1.44*** | 521 | *NifE* |
| gene1383 | asr1409 | 0.65 | 0.75 | 0.10 | 203 | *Nift* |

Note: “Inf” means the gene expression of control samples is absent. Nitrogenase gene, *Nif*; *, differently expressed gene with 1 < |Log_2_FC| < 3; **, highly differently expressed gene with Log_2_FC ≥ 3. In three paired comparisons, X vs. Y refers to Y/X.
