# Supplementary material for: Vegetative cells may perform nitrogen fixation function under nitrogen deprivation in Anabaena sp. strain PCC 7120 based on genome-wide differential expression analysis
Source: PLoS One. 2021 Mar 4;16(3):e0248155. doi: 10.1371/journal.pone.0248155 (PMC7932525; doi:10.1371/journal.pone.0248155)
Supplement: S5 Table — (DOCX) [file pone.0248155.s007.docx]

S5 Table. Primers used for qRT-qPCR

| Primer name | Sequence |
| --- | --- |
| *RnpA*_F | 5' -TTGCGAGCATTAAAACCGTC 3' |
| *RnpA*_R | 5'- TACGCACCACCGCCCTCT -3' |
| *Gvp*_F | 5'- AGCCAAACAGCAAGCGCAACAGC- 3' |
| *Gvp*_R | 5'- TGCTTTGGCTTGCTCAGTTCTGTTTTTT -3' |
| *NifH*_F | 5' -AGGTTGCGCCGGTCGTGGTATCA -3' |
| *NifH*_R | 5'- CCGCGAGCGATGTTGTTAGCAGCA- 3' |
| *PsaA*_F | 5'- CCGGTGCAGTTGTTCCATCCTCCA- 3' |
| *PsaA*_R | 5'- CCGGAGATACCCAAGGGCATACCA- 3' |
| PsaE_F | 5' -CGCGATCCTCAATGGGGCAACCTAGA -3' |
| PsaE_R | 5' -CTGGGCGATAGGCGGGTAGGTTGTT- 3' |

Note: F, forward primer; R: reverse primer. *Gvp*: Gas vesicle synthesis protein gene; *NifH*: nitrogenase iron protein gene; *PsaA*, PSI-A core protein gene of photosystem I; *PsaE*: PSI-E subunit of photosystem I.
